# Robust testing of haplotype/disease association

**DOI:** 10.1186/1471-2156-6-S1-S69

**Published:** 2005-12-30

**Authors:** Andrew S Allen, Glen A Satten

**Affiliations:** 1 Department of Biostatistics and Bioinformatics, Duke University, Durham, NC, USA; 2 Duke Clinical Research Institute, Duke University, Durham, NC, USA; 3 Centers for Disease Control and Prevention, Atlanta, GA, USA

## Abstract

Haplotypes, the combination of closely linked alleles that fall on the same chromosome, show great promise for studying the genetic components of complex diseases. However, when only multilocus genotype data are available, statistical approaches need to be employed to resolve haplotype phase ambiguity. Recently, we have proposed an approach to testing and estimating haplotype/disease association that is invariant to any existing genetic structure in the population. Here we evaluate this approach by applying it to the Genetic Analysis Workshop 14 simulated data.

## Background

Single-nucleotide polymorphism (SNP)-based haplotype association studies show great potential for dissecting genetic influences on complex diseases. Haplotype-based methods incorporate linkage disequilibrium information from many markers and, hence, may be more powerful than traditional linkage disequilibrium methods that focus on a single SNP. Haplotype-based methods also have the promise of being able to identify unique segments of DNA containing sequences predisposing individuals to disease. In addition, when multiple alleles at a single disease locus influence disease susceptibility, single marker tests can be under-powered relative to haplotype-based association methods [1]. In the Genetic Analysis Workshop 14 (GAW14) simulated dataset, multilocus genotypes were constructed using haplotypes at 2 of the 4 disease loci.

A major problem when conducting haplotype studies is that the available marker genotype data (often SNPs) are unphased, resulting in haplotype ambiguity. This fact has stimulated the development of a number of statistical techniques designed to reconstruct haplotype phase. Most of these methods treat the haplotypes as missing data and apply the expectation-maximization (EM) algorithm [2] to infer haplotype frequencies by assuming these frequencies are in Hardy-Weinberg equilibrium (HWE) [3-5]. This assumption results in estimates and tests that are sensitive to the genetic structure of the sampled population, potentially leading to biased estimates and incorrect inferences [6]. Recently, we have developed an approach to testing haplotype/disease association that does not vary based on the distribution of haplotypes in the population [7]. In this work, we give an overview of this approach and apply it to triad datasets extracted from the GAW14 simulated data.

## Methods

### Model and projection approach

We assume a case-parent sampling design in which individuals with disease (*D *= 1) or trait of interest and their parents are sampled. At a locus of interest, let the proband's genotype be denoted *G*_*o *_and let *G*_*p *_denote the parents' genotypes. Let *O *=(*G*_*o*_, *G*_*p*_) denote the observed data (realizations are denoted by *g*_*o*_, *g*_*p*_, *o*). Let the proband's haplotypes be denoted *H*_*o *_and let *H*_*p *_denote the parents' haplotypes (again, realizations are denoted by *h*_*o*_, *h*_*p*_). As a first step, note that we can write the *i*^th ^family's contribution to the observed-data likelihood as



where *γ *(*q*-dimensional) is the haplotype relative risk parameters of interest, *η *(*r*-dimensional) is nuisance parameters describing the distribution of parental haplotypes, and H(*o*) is the set of all *h*_*o*_, *h*_*p *_consistent with *o*. The model for Pr(*H*_*o*, *i *_= *h*_*o *_| *H*_*p*,*i *_= *h*_*p*_, *D *= 1;*γ*)| can be parameterized in a manner analogous to genotype relative risk models for case-parent designs [8]. We assume this model is correct (see below). This assumption is unnecessary when testing against the simple null hypothesis that no haplotype affects disease, but is required for valid parameter estimation or for tests that have a composite null hypothesis. The model for Pr(*H*_*p*, *i *_= *h*_*p *_| *D *= 1;*η*) is problematic since: 1) a nonparametric model (i.e., saturated multinomial) is both computationally challenging and nonidentifiable, and 2) an incorrectly specified parametric model (i.e., HWE) can lead to significant bias in parameter estimates.

Our approach is to find the efficient score (*U*) for the parameter of interest by projecting the observed data score onto the nuisance tangent space [9] assuming a saturated multinomial model for the distribution of parental haplotypes (note: HWE is not assumed). Computation of *U *is complicated by the fact that one must estimate Pr(*H*_*p*, *i *_= *h*_*p *_| *D *= 1;*η*) and the nuisance parameter, *η*, is not identifiable in the saturated multinomial model. However, we have shown that *U *has mean zero regardless of the model used to estimate Pr(*H*_*p*, *i *_= *h*_*p *_| *D *= 1;*η*) in the construction of the projection [7]. Hence, one can use an identifiable (but possibly incorrect) parametric model to estimate Pr(*H*_*p*, *i *_= *h*_*p *_| *D *= 1;*η*) and use this estimate to compute *U*, secure in the knowledge that *U *will still have mean zero. This leads to tests and estimators of haplotype effects that are robust to the misspecification of the distribution of parental haplotypes. Note that both parameter estimates and their estimated variances are robust to misspecification of the distribution of parental haplotypes. Further, if the parental haplotype distribution is correctly specified, these estimators and tests will be optimal, having minimum variance among all estimators that are robust to misspecification of the parental haplotype distribution (for complete details see [7]).

Parameter estimates using our model depend on correct specification of the haplotype relative risk model Pr(*H*_*o*, *i *_= *h*_*o *_| *H*_*p*, *i *_= *h*_*p*_, *D *= 1;*γ*). However, the availability of unbiased tests and estimators makes it possible to select a model that fits the observed data without having to specify correctly the parental haplotype distribution. In one special situation, our tests (but not estimators) are robust to misspecification of the relative risk model: when testing the simple "global" null hypothesis that no haplotype affects disease risk. In this case, correct specification of the risk model only affects the power of the test, not its size.

### Application to GAW14 data

We applied this method to a hypothetical candidate gene study in which triads were ascertained from replicates 1–10 by the presence of subclinical phenotype c in an offspring. Only triads in the Aipotu, Karangar, and Danacaa populations were sampled (one triad per nuclear family). The genomic region and phenotype of interest were guided by consulting the answers throughout our analyses. For each replicate a region of the genome spanned by markers B09T8321–B09T8360 (known to contain trait locus D4 as well as linkage disequilibrium) was analyzed. Our goal was to study the effects of varying haplotype size on localization of a disease locus as we scanned across a region known to contain a disease locus. Because the D2 locus (the only other trait locus in a region of linkage disequilibrium) was at the end of a chromosome beyond the last visible SNP, we did not analyze on this region. We fit a multiplicative haplotype relative risk model in which we included a parameter for all haplotypes with frequencies greater than 5%. The haplotype with the highest frequency was used as a reference. The resulting score test is invariant to this reference choice, but parameter interpretation would be affected. We report -log_10_(*p*-values) derived by an overall score test formed by evaluating the efficient score at *γ *= 0. Note the degrees of freedom for our test varied both with the number of SNPs per haplotype and the specific loci included, depending on how many haplotypes had frequency greater than 5%.

## Results

**Figure 1:**
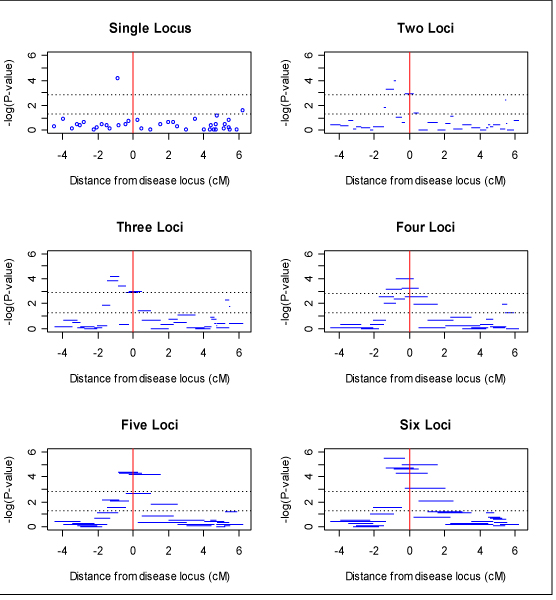
**Result of overall haplotype score test with windows of 1 to 6 loci**. Upper and lower horizontal lines correspond to Bonferroni-corrected and uncorrected 0.05 significance levels, respectively. Vertical line corresponds to location of disease locus.

Figure 1 presents the results of haplotype analyses of data replicate 1 using haplotype windows of 1 through 6 loci. Analyses with longer haplotypes seem to have higher -log_10_-transformed *p*-values with the 6 loci analysis having an order of magnitude smaller *p*-values than the single locus analysis. Interestingly, this increase in -log_10_-transformed *p*-values in the region of the disease loci was also accompanied with a suppression of a secondary (false) peak in the region of markers 30–40. It also appears that the analyses of longer haplotypes localize the disease locus better than the single locus analysis.

**Figure 2:**
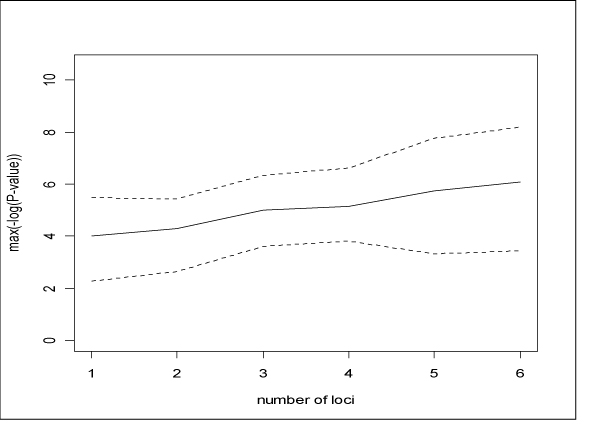
**Maximum (across region within replicate) of -log_10_-transformed *p*-values**. Solid line: Mean value (over replicates). Upper and lower dotted lines: 80^th ^percentile and 20^th ^percentile of the maximum -log_10_-tranformed *p*-values, respectively.

These are observations on a single simulated dataset. To determine a general pattern, we analyzed 9 other replicate datasets (datasets 2–10). Figure 2 presents a summary of the -log_10_-transformed *p*-values by size of analysis window for all 10 datasets. The solid line denotes the mean value of the maximum -log_10_(P-value) (maximum is taken over the region). The upper and lower dotted lines represent the 80^th ^and 20^th ^percentiles of maximum -log_10_-transformed *p*-values, respectively. Larger analysis haplotype size does seem to lead to increased -log_10_-transformed *p*-values. We confirmed this trend statistically by using generalized estimating equations (GEE) to regress maximum -log_10_-transformed *p*-values on the size of the haplotype window (GEE was used to account for correlation between analyses on the same dataset). We found that the trend for larger haplotype windows to have larger maximum -log_10_-transformed *p*-values, shown in Figure 2, was significant (*p *< 0.0001).

**Figure 3:**
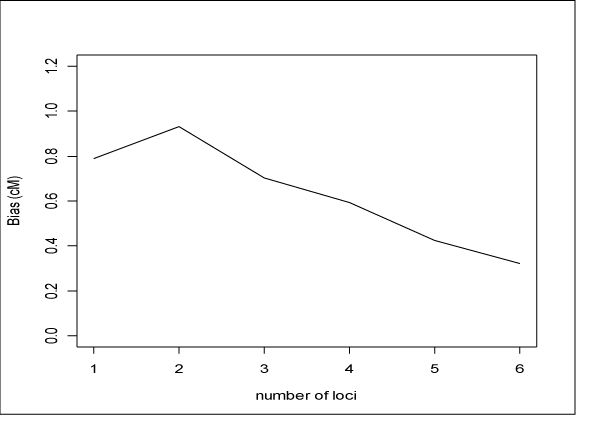
**Bias in disease locus localization**. Average distance to disease locus of average (over region) of significant (Bonferroni-corrected) loci.

We also investigated the effect of haplotype size on disease locus localization. For each replicate we averaged the locations of significant marker loci (or midpoint of significant haplotypes) and then computed distance (cM) between this average and the disease locus. We then averaged this bias over the replicated datasets. The results of this exercise are presented in Figure 3.

In the simulated datasets we analyzed, use of larger haplotypes does seem to better localize the disease locus. Again, we confirmed this trend by using GEEs to regress the average bias of significant loci on the size of the haplotype window. We found that the trend for larger haplotype windows to exhibit smaller bias, illustrated in Figure 3, was significant (*p *= 0.0141).

## Conclusion

Though we attempted to approximate candidate gene studies of the various phenotypes, it is difficult to interpret the results because the data were certainly not generated with this intent in mind. For example, the haplotypes structure was only used to generate linkage disequilibrium and was not specifically related to phenotype. Nevertheless, the analyses we conducted do suggest a few patterns that merit further exploration. Analyses of longer (more SNPs) haplotypes seem to both result in smaller *p*-values in the disease region relative to single locus analyses and result in better localization of the disease loci. In addition, the longer haplotype analyses seemed to suppress secondary (false) peaks, resulting in fewer false positive signals.

## Abbreviations

EM: Expectation maximization

GAW14: Genetic Analysis Workshop 14

GEE: Generalized estimating equations

HWE: Hardy-Weinberg equilibrium

SNP: Single-nucleotide polymorphism
